# Overcoming the growth–infectivity trade‐off in a bacteriophage slows bacterial resistance evolution

**DOI:** 10.1111/eva.13260

**Published:** 2021-06-19

**Authors:** Quan‐Guo Zhang, Xiao‐Lin Chu, Angus Buckling

**Affiliations:** ^1^ State Key Laboratory of Earth Surface Processes and Resource Ecology and MOE Key Laboratory for Biodiversity Science and Ecological Engineering College of Life Sciences Beijing Normal University Beijing China; ^2^ ESI & CEC Biosciences University of Exeter Penryn UK

**Keywords:** bacteriophage therapy, biological control, evolutionary training, experimental evolution, resistance evolution, trade‐off

## Abstract

The use of lytic bacteriophages for treating harmful bacteria (phage therapy) is faced with the challenge of bacterial resistance evolution. Phage strains with certain traits, for example, rapid growth and relatively broad infectivity ranges, may enjoy an advantage in slowing bacterial resistance evolution. Here, we show the possibility for laboratory selection programs (“evolutionary training”) to yield phage genotypes with both high growth rate and broad infectivity, traits between which a trade‐off has been assumed. We worked with a lytic phage that infects the bacterium *Pseudomonas* *fluorescens* and adopted three types of training strategies: evolution on susceptible bacteria, coevolution with bacteria, and rotation between evolution and coevolution phases. Overall, there was a trade‐off between growth rate and infectivity range in the evolved phage isolates, including those from the rotation training programs. A small number of phages had both high growth rate and broad infectivity, and those trade‐off‐overcoming phages could slow or even completely prevent resistance evolution in initially susceptible bacterial populations. Our findings show the promise of well‐designed evolutionary training programs, in particular an evolution/coevolution rotation selection regime, for obtaining therapeutically useful phage materials.

## 1 INTRODUCTION

The crisis of antibiotic resistance has led to a resurgent interest in the use of bacteriophages (phages) to treat bacterial infections (Alisky et al., 1998; Barrow & Soothill, 1997; Biswas et al., 2002; Bull et al., 2002, 2010; Chanishvili et al., 2001; Kutateladze & Adamia, 2010; Levin & Bull, 1996; Monk et al., 2010; Pirnay et al., 2011; Summers, 2001). However, resistance evolution is also a major consideration in phage therapy, especially given that bacteria can evolve resistance to phages in a matter of days or even hours. Therefore, significant therapeutic benefits would be gained if better‐designed phage applications can slow or even prevent resistance evolution in bacterial infections (Chan et al., 2013; Pirnay et al., 2011; Torres‐Barceló, 2018).

Intrinsic properties of the bacteriophage combinations have a major impact on the rate of resistance evolution (Bohannan & Lenski, 2000; Lenski & Levin, 1985). In particular, different resistance mechanisms may vary in the likelihood of occurrence, fitness costs, and whether phages can evolve counter defense mechanisms (Buckling & Brockhurst, 2012; Labrie et al., 2010). The efficacy of phage treatments can be enhanced by using combinations of phages that can overcome different resistance mechanisms (Brockhurst et al., 2017; Chan & Abedon, 2012; Gu et al., 2012; Hall et al., 2012; Loc‐Carrillo & Abedon, 2011; Wright et al., 2019). Using phages with life‐history traits that result in high net phage growth rates, and hence lower bacterial densities, is also desirable (Cairns & Payne, 2008; Torres‐Barceló, 2018).

Natural phages are unlikely to be optimized for phage therapy. Their environments differ in many ways from the therapeutic context, and natural selection may not favor phage traits desirable for bacterial elimination. Experimental adaptation of phages to host bacteria, or “evolutionary training,” has thus been suggested as a means to increase the effectiveness of phage therapy (Rohde et al., 2018). There have been two major approaches. First, repeated propagation of a phage on a susceptible bacterial strain can improve phage growth performance on certain host bacterial genotypes (Betts et al., 2013; Morello et al., 2011). Second, coevolving phage with bacteria can result in phage strains that are harder to evolve resistance to (Friman et al., 2016), presumably because the coevolved phages can infect a wider range of bacterial genotypes with different resistance mechanisms (Forde et al., 2008; Poullain et al., 2008).

Here, we investigate the potential for single‐phage isolates to evolve both high growth rate and broad infectivity range, and the consequences for affecting bacterial resistance evolution. Previous work indicates that phages generally trade off broad infectivity against maximum growth rate on hosts that can be infected (de Jonge et al., 2019; Frickel et al., 2016; Keen, 2014; Poullain et al., 2008). This finding is consistent with the commonly reported fitness costs associated with niche generalism in a range of taxa (Bell, 2008; Bono et al., 2017; Henry et al., 2008; Kassen, 2002, 2014; Roff & Fairbairn, 2007; Whitlock, 1996). However, populations, particularly those evolving in temporally varying environments, can sometimes become generalists with little apparent costs (Bennett & Lenski, 2007; Bono et al., 2017; de Vos et al., 2015; Karve et al., 2016; Sachdeva et al., 2020; Sexton et al., 2017).

**FIGURE 1:**
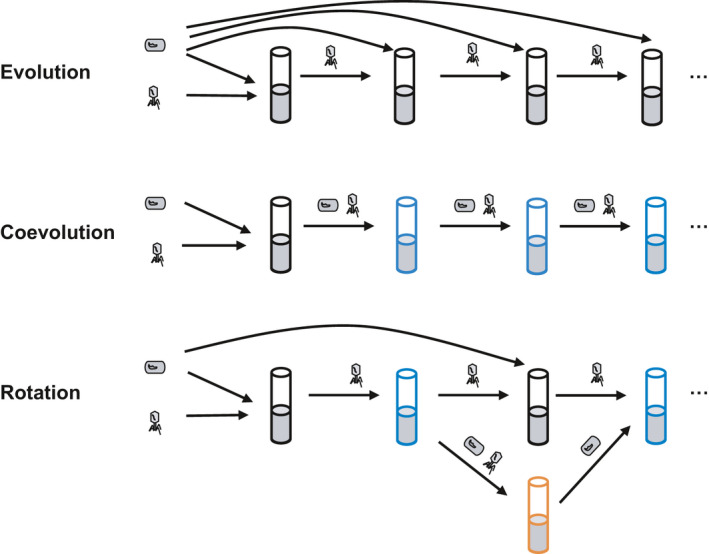
Graphical illustration of the phage training strategies. Black tubes show where phages were propagated on the ancestral, susceptible, bacteria. Blue tubes are where phages coevolved with bacteria. Orange tubes show where cultures were incubated at a high temperature that could prevent phage growth without harming the bacteria (and thus isolate bacteria from cultures)

We experimentally evolved a bacteriophage (lytic phage SBW25Φ2) on the host bacterium *Pseudomonas* *fluorescens* SBW25 to determine whether it is possible to overcome the growth–infectivity trade‐off. Such a trade‐off was observed in previous work that compared phages replicating repeatedly on the susceptible host strain only (“evolution”) and those coevolving with bacteria (“coevolution”) (Poullain et al., 2008). Here, we considered those two selection scenarios as well as a novel selection regime “evolution/coevolution rotation,” switching between “evolution” and “coevolution” with different temporal combinations (Figure 1). We then determined how the evolved infectivity and growth rate traits may affect bacterial resistance evolution, and the genetic basis for the evolved phage traits. We note that that *P*.* fluorescens* is unlikely to be a target of phage therapy, and we use this system because of its extensive use in studies of bacteria–virus coevolution (Buckling & Brockhurst, 2012).

## 2 MATERIALS AND METHODS

### 2.1 Strains and culture conditions

The bacterium *P*. *fluorescens* SBW25 (Rainey & Bailey, 1996) and its lytic bacteriophage virus SBW25Φ2 (Buckling & Rainey, 2002) were used in this study. Cultures were grown in M9KB medium (M9 salt solution supplemented with 10 g L^−1^ glycerol and 20 g L^−1^ proteose peptone no. 3) at 28°C, without shaking. Isolation of phages from cultures was achieved by mixing 1 ml of culture with 100 µl of chloroform, which was then vortexed to lyse the bacterial cells, and centrifuged at 15,800 *g* for 2 min to pellet the bacteria debris, leaving a suspension of phage in the supernatant. Phage density was measured by plating soft agar medium (M9KB medium with 7 g L^−1^ agar) with phage dilutions and ancestral bacterial cells, and counting the number of plaque‐forming units (PFUs) after 24‐h incubation at 28°C. Culture samples were stored at −80°C in 40% (vol/vol) glycerol.

### 2.2 Phage selection experiment

Cultures were grown in 5 ml of nutrient medium in 30‐ml centrifuge tubes with loosened lids, without shaking. Every selection line was initially inoculated with approximately 1.5 × 10^7^ cells of the ancestral bacterium (5 μl of bacterial culture that had been reconditioned for 2 days) and 10^4^ particles of the ancestral phage. Populations were propagated for twelve 2‐day serial transfers. Five selection treatments were set up: one “evolution,” one “coevolution” and three “evolution/coevolution rotation” treatments, with eight replicates for each treatment. In the “evolution” treatment, phage populations were isolated from cultures at every transfer and 5 μl of the phage extracts were added into fresh microcosms with 5 μl of the ancestral bacterial culture. In the “coevolution” treatment, 5 μl of each culture containing both bacteria and phages was transferred to a fresh microcosm at each passage. In the “evolution/coevolution rotation” treatments, each phage population coevolved with bacteria for a certain number of transfers (coevolution phase). Phages were isolated and 5 μl of phage extract was added into a fresh microcosm with 5 μl of the ancestral bacterial culture, for one new transfer of propagation (evolution phase). Meanwhile, 5 μl of the old culture containing both bacteria and phages were added to another microcosm and incubated for one transfer at 31°C which could stop phage growth but did not harm bacterial growth (Padfield et al., 2020; Zhang & Buckling, 2011). Phages isolated from the evolution‐phase microcosm were added into a fresh microcosm with bacteria that had coevolved with them previously and experienced the 31°C incubation (phages undetectable in cultures treated by 31°C), such they could “reunite” and continue their coevolutionary interactions (Figure 1). Three rotation treatments were set up: switching between 1‐transfer evolution phase and 1‐, 2‐, or 3‐transfer coevolution phases; and they were referred to as “rotation A,” “rotation B,” and “rotation C,” respectively. The “Rotation” regime shown in Figure 1 was, specifically speaking, “rotation A” in our study. Note that removal of phages from coevolution‐phase cultures could also be achieved by other methods such as Virkon treatment (Morgan et al., 2005; see a more detailed discussion about phage removal in Appendix S1).

Eight out of the 40 phage selection lines went extinct during the selection experiment. One phage isolate was randomly chosen from each of the 32 surviving phage populations, by picking one plaque from the bacterial lawn used for phage density measurement.

### 2.3 Phage traits measurement

Growth rate of phage isolates was measured as the number of doublings within a 4‐h incubation with ancestral bacterial cells at a low multiplicity of infection (MOI). Approximately 10^4^ phage particles were added to 5 ml of nutrient medium with 1.5 × 10^7^ ancestral bacterial cells of stationary phase, and incubated for 4 h. Initial and final phage densities (*N*_0_ and *N*_t_) were measured, and the number of doublings was calculated as log_2_(*N*_t_/*N*_0_). Infectivity ranges were measured against 32 reference bacterial isolates chosen from the selection lines of “coevolution” and “rotation” treatments (one colony randomly picked up from each of these selection lines), which were all resistant to the ancestral phage. To examine the infectivity of a phage isolate, culture of each of the reference bacterial isolates was streaked across a line of phage that had previously been streaked and dried on agar plates (20 µl of phage culture for each streak), and incubated for 24 h. Inhibition of bacterial growth indicated phage infectivity. Infectivity range of each phage isolate was defined as the proportion of susceptible bacterial isolates among the total of 32.

### 2.4 Success of phages in slowing bacterial resistance evolution

All the 32 phage isolates were assayed for their effect on controlling growth of initially susceptible bacterial populations. Each phage isolate was grown with the ancestral bacterial cells in 220 μl of nutrient medium, in 96‐well microplates. The initial phage and bacterial concentrations were 3 × 10^2^ and 3 × 10^6^ ml^−1^, respectively. We intentionally used a low MOI for the assays because low‐dosage use is desirable in phage applications (Loc‐Carrillo & Abedon, 2011). Each phage was assayed in ten replicate cultures. The cultures were grown in a static incubator for 7 days, during which optical density values were measured every day. A bacterial population becoming turbid (specifically, OD >0.05) indicated the failure of a phage to keep the bacteria in check. Cultures that were still clear at the end of the 7‐day assay were spread onto agar plates; counting colony‐forming units confirmed that those clear cultures contained no viable bacterial cells. We calculated the frequencies of complete bacterial clearance for each phage isolate.

When phages failed to drive bacteria extinct, the phage isolates varied in how soon the control effect on bacterial growth failed. We calculated a “duration of effective control” score for each phage isolate, with bacteria‐extinct assays excluded from estimating this index. The first day that a culture became turbid was considered as the failure date for the phage in that particular assay culture. We used a parametric survival regression model, “survreg” function provided by R package “survival” (Therneau, 2020), to estimate the mean failure time for each phage (referred to as “duration of effective control”).

### 2.5 Sequence analysis

High‐concentration phage suspensions were prepared for DNA extraction. To this end, we spread pure phage cultures over soft agar medium containing the ancestral bacterial cells. After confluent lysis was observed, 10 ml of SM buffer (50 mM Tris‐HCl of pH 7.5, 100 mM NaCl, and 8 mM MgSO_4_) was added to each plate, further incubated overnight at 4°C. The supernatant was collected, and the phage was isolated by chloroform treatment (Scanlan et al., 2011). DNA extraction was carried out using Norgen's phage DNA isolation kit. Library construction and resequencing were conducted at Personalbio using the Illumina MiSeq platform. We obtained 250‐bp paired‐end reads for all samples; and sequencing resulted in an average of 6,019,391 reads per sample. Removal of adapter sequences and quality trimming were carried out using Trimmomatic version 0.39 (Bolger et al., 2014). Leading and trailing bases with quality below 3 were removed. Sequence quality was assessed for each read in a 5‐bp sliding window; and reads were discarded when Phred33 quality score fell below average of 20 within the window. Reads with a length below 50 were excluded from the analysis. Trimmed reads were mapped to the reference genome (EMBL accession FN594518) with bwa‐mem algorithm version 0.7.12 (Li & Durbin, 2010). Base‐pair substitutions (BPSs) and indels were called with samtools mpileup version 0.1.9 (Li et al., 2009). The sites whose read depth was less than 30× were excluded to minimize the impact of sequencing errors. BPSs and indels were annotated to the reference genome and classified as intergenic and genic (which further classified as synonymous, nonsynonymous for BPSs; and nonframeshift, frameshift, startloss for indels) using Annovar version 2019Oct24 (Wang et al., 2010). Our ancestral phage strain differed from the reference genome in one synonymous BPS, which was also shared by all our evolved phage isolates; this was excluded in subsequent analysis.

### 2.6 Statistical analysis

Analyses were carried out in the R environment. The existence of an overall trade‐off between growth rate and infectivity was analyzed using Pearson's correlation test, which turned out to be significantly negative. Differences among selection treatments in growth or infectivity were analyzed using ANOVA and subsequent stepwise model simplification that grouped treatments with no significant difference (Crawley, 2013); and R scripts for analysis were available in Appendix S2. Being aware of an overall growth–infectivity trade‐off across our phage isolates, we investigated the possibility that a small number of exceptional, trade‐off‐overcoming, phages existed. We screened phage isolates for those that showed large deviation from the overall growth–infectivity relationship. This was done by detecting outlier samples in principal component analysis (PCA) for growth and infectivity, where the outliers detected were those with large overall residuals in both variables. PCA was performed using the “prcomp” function, and outlier samples were recognized based on the principal component (PC) scores calculated for each sample (Appendix S2).

We analyzed whether growth rate and infectivity range were significant predictors of the “duration of effective control” using a general linear model. No formal statistical analysis was performed for how the probability of bacterial extinction responded to phage growth rate and infectivity, as the data had an extremely biased distribution (two positive values and 30 zero values).

The association between mutations and phage traits was analyzed based on the incidence of mutations. The median of growth rate values was used as a cutoff value to categorize phage isolates into high‐ and low‐growth rate groups. We determined whether the number of hits of each mutation in the 16 high‐growth rate phages significantly differed from that in the 16 low‐growth rate phages, using Exact test provided by R package “Exact” (Calhoun, 2020). The same analyses were done for the possible association between mutations and infectivity range.

## 3 RESULTS

### 3.1 The growth–infectivity trade‐off

**FIGURE 2:**
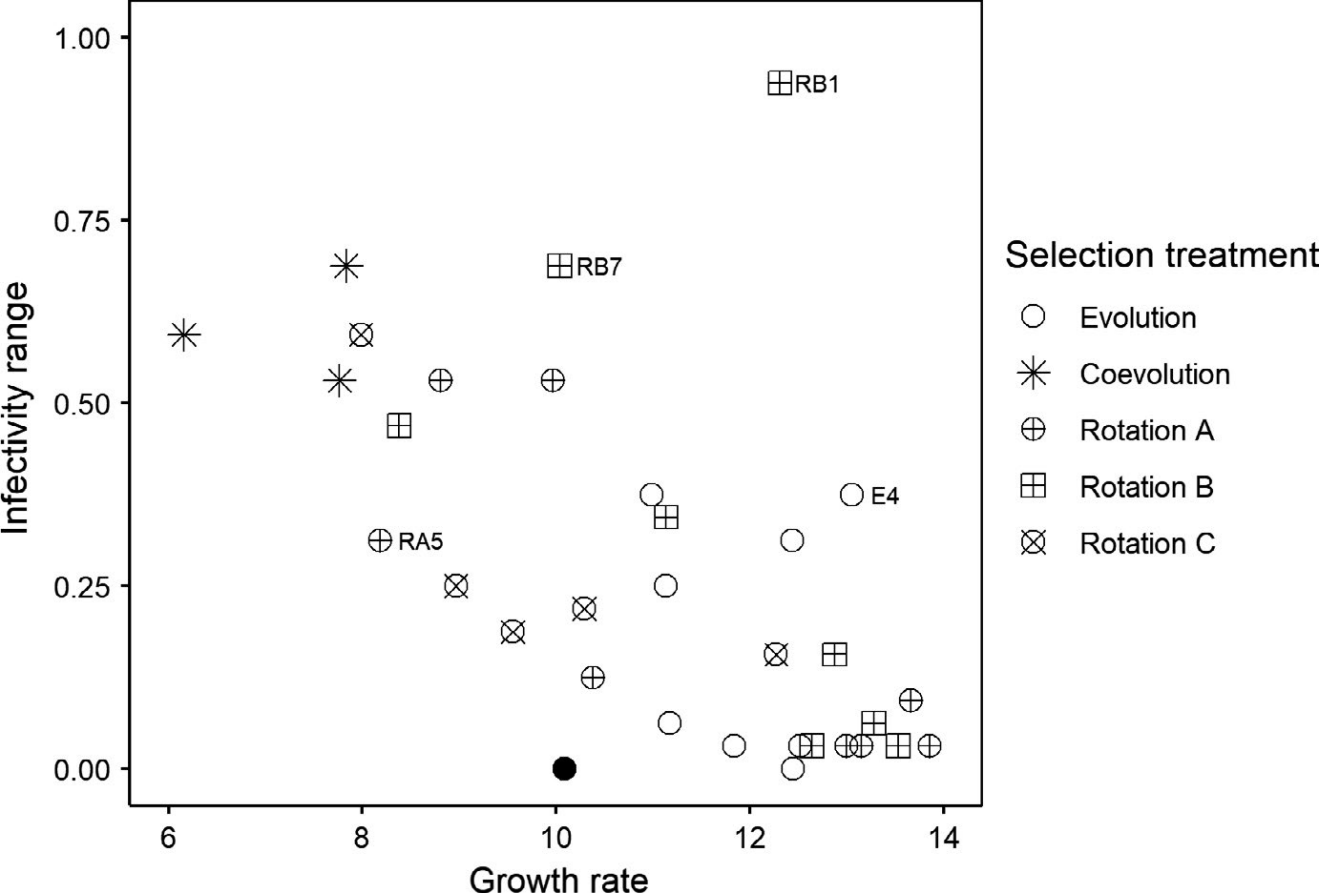
Growth rate and infectivity range of phage isolates from the selection experiment, grouped by selection regime. Data points showing large deviation from the overall growth–infectivity relationship are annotated with phage isolate ID. The ancestral phage strain is shown here (filled circle) but not included in data analysis

A total of 40 phage populations were evolved under five selection treatments: one “evolution” (E), one “coevolution” (C), and three “evolution/coevolution rotation” regimes. Among the three rotation regimes, “rotation A” (RA) had equal lengths of evolution and coevolution phases of selection, while “rotation B” (RB), and particularly “rotation C” (RC), had a longer coevolution phase of selection. Five “coevolution” phage lines and three “rotation C” phage lines went extinct. Phage lines under the other three selection treatments all persisted through the experiment (5/8 significantly larger than 0/8, Exact test, *p* = 8.06 × 10^−3^; 3/8 marginally non‐significantly larger than 0/8, *p* = 0.0823). One random phage isolate was selected from each of the 32 surviving populations. There was a negative correlation between growth rate and infectivity among those phage isolates, suggesting a trade‐off between the two traits (Figure 2; Pearson's correlation test, *r* = −0.618, *df* = 30, *p* = 1.62 × 10^−4^). Note that the ancestral phage strain was not included in the analysis, as phenotypes are likely to be affected by general adaptation to the laboratory environment.

The “coevolution” phages had a significantly lower growth rate and larger infectivity range than the “evolution” phages (Figure 2; Table S1). Phages from “rotation A” and “rotation B” treatments did not significantly differ from “evolution” phages in mean growth rate and infectivity. The average growth rate of “rotation C” phages lays between those of “evolution” and “coevolution” phages, while their mean infectivity value did not differ from “evolution” phages (Table S1).

### 3.2 The existence of trade‐off‐overcoming phages

Outlier phage samples in growth and infectivity values were identified with PCA analysis. Four outliers (phage E4, RA5, RB1, and RB7) were identified using a criterion that difference between a PC score and the median was greater than 1.75 times of the median of absolute deviation, and two (E4 and RB1) were recognized when the threshold value was set as 2 times. Three of those outliers had positive residuals (i.e., growth and infectivity values greater than predicted by the model): one from “evolution” treatment (phage isolate ID: E4) and two from “rotation B” treatment (RB1 and RB7); and those three were hereafter referred to as trade‐off‐overcoming phages. One phage in particular (RB1) had both growth and infectivity close to that of the maximum values across all phage isolates. An alternative analysis approach based on linear regression between growth and infectivity and subsequent model checking identified the same three trade‐off‐overcoming phages (Appendix S1; Figure S1).

### 3.3 Impacts on bacterial resistance evolution

**FIGURE 3:**
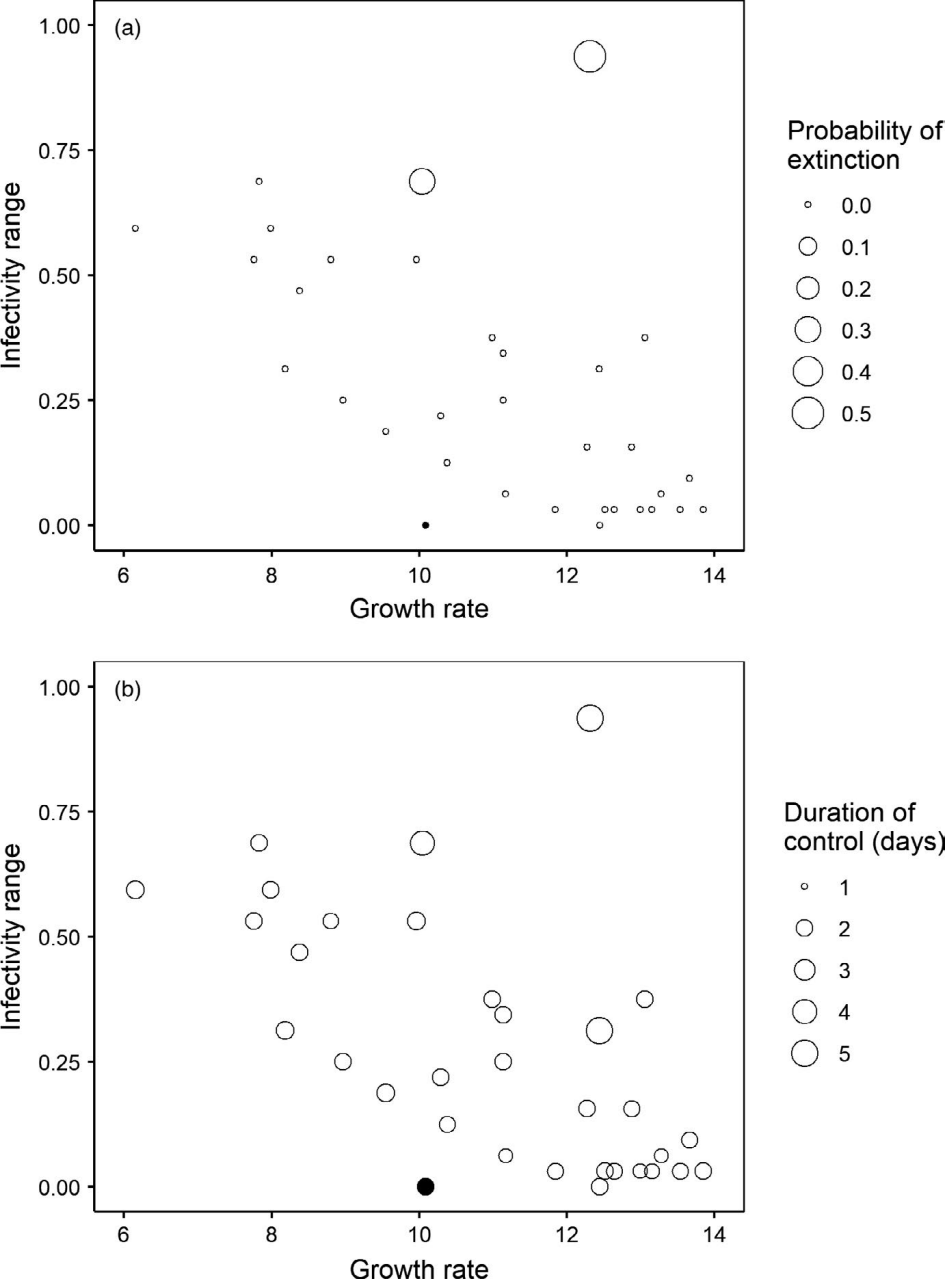
Two estimates of the success of phages in slowing resistance evolution in initially susceptible bacterial populations mapped to point size on the growth–infectivity plot. (a) The probability to drive bacteria extinct. (b) The “duration of effective control” (the period of time before bacteria evolved resistance and showed obvious growth) in assays where phages failed to drive bacterial extinct. The ancestral phage strain is shown here (filled circle) but not included in data analysis

When used to treat initially susceptible bacterial populations, two phage isolates, RB1 and RB7, drove bacteria extinct at frequencies of 5/10 and 3/10, respectively; and all the other phages failed to prevent bacterial resistance evolution (Figure 3a). In cases where phages failed to drive bacteria extinct, the phage isolates varied in how soon the control effect on bacterial growth failed. The “duration of effective control” was longer when phages had greater growth rates or broader infectivity ranges (Figure 3b; general linear model, Type II tests, growth, *F*_1,29_ = 10.414, *p* = 3.10 × 10^−3^; 0; infectivity, *F*_1,29_ = 26.0, *p* = 1.95 × 10^−5^; interaction term non‐significant and removed by model simplification).

### 3.4 Possible genetic basis of phage growth and infectivity

On average, each phage isolate had 2.56 (±0.373; SE) genic‐region mutations, including both base‐pair substitutions and indels. Two mutations in a single gene, *SBWP25_0036* (encoding a phage tail fiber protein), had relatively high incidence in our phage isolates, A35898G with a total of 15 hits and G36794A with 14 hits (Table S2). Mutation A35898G showed a positive association with broad infectivity and a negative association with high growth rate. Mutation G36794A had a positive association with broad infectivity, and its incidence did not differ between high‐ and low‐growth phages; moreover, this mutation was observed in all the three trade‐off‐overcoming phages (Table S2). The other mutations had relatively low incidences in our phage isolates (≤4 total hits), and none of them showed a significant association with high growth rate or broad infectivity. However, three mutations showed an association with trade‐off‐overcoming phages: synonymous mutation A1708G in gene *SBWP25_0003* (hypothetical protein) and nonsynonymous mutation A27836C and A28991G in gene *SBWP25_0032* (encoding a phage tail tubular protein; Table S2).

## 4 DISCUSSION

Our evolutionary training programs resulted in a small number of phage strains that could overcome the growth–infectivity trade‐off. Two out of the three trade‐off‐overcoming phages (RB1 and RB7) were sometimes able to completely prevent bacterial resistance evolution, driving bacteria extinct; and all the other phages failed to do so (Figure 3a). Even in replicate assays where bacteria survived, phage RB1 and RB7 had longer “duration of effective control,” that is, they slowed bacterial resistance evolution (Figure 3b). The “duration of effective control” period was longer in bacterial cultures treated with phages with faster growth or broader infectivity (Figure 3b). Two out of the three trade‐off‐overcoming phages were from the “rotation B” selection treatment (alternating between 1‐transfer evolution and 2‐transfer coevolution), and they were the only “super‐phages” that could prevent bacterial resistance evolution (Figure 3). This suggests that well‐designed rotation training selection could be particularly helpful for obtaining therapeutically useful phage strains.

Analysis of phage sequences identified two mutations with significant association with broad infectivity, A35898G and G36794A (Table S2). The two mutations were in a single gene, *SBWP25_0036*, which was shown by earlier studies as the most important gene for infectivity changes in this phage (Paterson et al., 2010; Scanlan et al., 2011). Mutation A35898G showed a negative association with high growth rate, while the other mutations, including G36794A, did not show any significant association with growth rate. It is possible that the infectivity change by mutation G36794A did not incur fitness costs in growth rate at all or that the fitness costs have been compensated for by other mutations. Mutation G36794A was observed in all the three trade‐off‐overcoming phages. We also found three further mutations associated with the trade‐off‐overcoming phages (A1708G, A27836C, and A28991G), but these did not show a significant association with high growth or broad infectivity across all the 32 phage isolates. These mutations may have contributed to compensatory adaptation to fitness costs associated with broad infectivity (see further discussion in Appendix S1).

Phages that can control bacterial hosts, or ideally drive them locally extinct, are desirable materials in clinical or agricultural settings. Such phage strains may not however be common in nature. The potential to eliminate host populations may be selected against as an evolutionary dead end for consumer species including phages, particularly in isolated local habitats, as the extinction of resources would be followed by the extinction of consumers themselves (Boots & Sasaki, 1999; Ewald, 1983; Kerr et al., 2006; Leggett et al., 2013). The evolution of very virulent phages could also be limited by genetic and physiological constraints, including trade‐offs involving fitness components in addition to growth and infectivity such as decay rate or thermostability (De Paepe & Taddei, 2006). Our proof‐of‐concept study demonstrates that well‐designed artificial selection may help to circumvent those evolutionary hurdles and obtain phage materials desirable for application purposes.

## CONFLICT OF INTEREST

None declared.

## Supporting information

Supplementary Material

Data S1

## Data Availability

Raw sequence data are available at NCBI Sequence Read Archive (BioProject accession number PRJNA688603), and other data are available in the supplementary material of this article (Data S1).
