# Supplementary material for: Overcoming the growth–infectivity trade‐off in a bacteriophage slows bacterial resistance evolution
Source: Evol Appl. 2021 Jun 19;14(8):2055–63. doi: 10.1111/eva.13260 (PMC8372119; doi:10.1111/eva.13260)
Supplement: Supplementary file 1 — Supplementary Material [file EVA-14-2055-s001.docx]

**Supplementary material**

**Appendix S1 Supplementary results and discussion**

*On the removal of phages from coevolution-phase microcosms*

One critical methodological aspect of our “evolution/coevolution rotation” selection treatment is to remove phages, or at least reduce phage density by a very large magnitude, in certain cultures (orange tubes in Fig. 1). This is to ensure that phage populations in “reunited” coevolution-phase cultures will be (mainly) contributed by those had experienced the evolution-phase treatment.

We used high-temperature treatment (31°C) to reduce phage density. Our phages fail to replicate when grown at temperatures > 30°C ([Zhang & Buckling, 2011](#_ENREF_8)). When incubated at 31°C, bacteria growth is not impacted; phages normally suffer a high decay rate and population density typically reduces by > 90%. In the present study, phages were not detectable (< 10^2^ ml^-1^) in every culture that had experienced the 31°C treatment, while densities of phage populations from the evolution-phase treatment were > 10^8^ ml^-1^. We did not expect that this very short (1-transfer) phage removal procedure would cause large-magnitude reduction in bacterial population resistance, as earlier studies suggested that phage extinction was not followed by immediate reversion of bacterial resistance ([Zhang & Buckling, 2016](#_ENREF_9)).

Viruses typically have lower thermostability compared with bacteria ([Danovaro et al., 2011](#_ENREF_1); [Tuladhar, Bouwknegt, Zwietering, Koopmans, & Duizer, 2012](#_ENREF_6); [Ulmanen, Broni, & Krug, 1983](#_ENREF_7)), therefore, the high-temperature treatment may reduce population sizes of many phage taxa. Using antiviral disinfectants may also reduce phage, relative to bacterial, densities, or even completely remove phages. One example is the chemical Virkon ([Lopez-Pascua & Buckling, 2008](#_ENREF_3); [Morgan, Gandon, & Buckling, 2005](#_ENREF_4)).

*An alternative approach for detecting outlier samples*

Detection of outlier samples in growth rate and infectivity range data was also carried out using linear regression between those two variables. Model checking of linear regression models can identify samples with very large residuals (in the response variable), which are highlighted in the “Residuals vs Fitted” plot. Two linear regression models were performed, the first with infectivity range as response variable and growth rate as explanatory variable, and the second with the opposite positions for the two variables. Slopes fitted by the two regression models were not exactly the reciprocal of each other; and model checking of the two models could identify different large-residual data points. Five outlier samples were recognized (Fig. S1; see R script in **Appendix** S2). Three out of the five outliers showed positive residual values and could be considered as putative trade-off-overcoming phages (phage ID: E4, RB1 and RB7).

***Traits of the ancestral phage***

**The ancestral phage had a growth rate of 10.1, and an infectivity range score of 0. Its success score in preventing bacterial resistance evolution was 0, and its “duration of effective control” score was 2.0 (days). Note that an infectivity score of 0 does not mean the failure to infect all strains of host bacteria. The infectivity score was estimated using bacterial colonies isolated from our experimental microcosms where bacteria had coevolved with phages. The ancestral phage can infect the ancestral bacterial strain.**

**If included in our analysis presented in Figure 2, the ancestral phage would be identified as an outlier sample with growth and infectivity values lower than expected. Therefore, the evolutionary changes of our phages are consistent with a scenario where trade-offs emerge with no, or weak, cost of adaptation (**[**Kassen, 2014**](#_ENREF_2)**). Specifically, evolved phages showed improved performance in one trait with no reduction, or only slight reduction, of performance in another trait, compared with the ancestral genotype. As different evolved genotypes may differ in which traits they show fitness gain, a trade-off among them can still emerge. The same pattern was observed in a previous study with the phage** SBW25Φ2, where “evolution” and “coevolution” selection regimes were compared **(**[**Poullain, Gandon, Brockhurst, Buckling, & Hochberg, 2008**](#_ENREF_5)**).**

***Further results and discussion about the*** *genetic basis of phage growth and infectivity*

On average, each phage isolate had 2.56 (± 0.373; SE) genic-region mutations. The number of mutations in phage isolates showed a significantly negative relationship with growth rate (general linear model, *F*_1,30_ = 8.81, *P* = 5.85 × 10^-3^), and a positive relationship with infectivity range (*F*_1,30_ = 14.2, *P* = 7.26 × 10^-4^). The three trade-off-overcoming phages had an average of 3.67 (± 0.667) mutations, compared with an average of 2.45 (± 0.402) in the remaining 29 phages (difference not statistically significant, possibly because of the very uneven sample sizes; ANOVA, *F*_1,30_ = 1.40, *P* = 0.246). An overall positive mutation number-infectivity relationship suggests that increased infectivity range generally involved the occurrence of a larger number of mutations in our phages. Meanwhile, the negative relationship between mutation number and growth rate implies that a large portion of mutations found in our phages may have impaired growth performance. Therefore, our ancestral phage may have a genetic background that allows for multiple evolutionary trajectories to increased infectivity, but a smaller chance for enhanced growth rate. If this is true, to overcome a growth-infectivity trade-off in this phage may involve compensation of fitness costs of those mutations driving infectivity changes. The observation of three mutations that are associated with trade-off-overcoming but not high growth or broad infectivity (A1708G, A27836C and A28991G) is consistent with this notion.

**Appendix S2 R script for data analysis**

# read data

data.training<-read.csv('E:\\data.temp\\phage.training.csv',header=T)

names(data.training)

# correlation test

cor.test(data.training$growth.rate,data.training$infectivity.range)

# ANOVA analysis for growth data

tapply(data.training$growth.rate,data.training$selection.treatment,mean)

tapply(data.training$growth.rate,data.training$selection.treatment,sd)

TreatA<-as.factor(data.training$selection.treatment)

modelA<-lm(data.training$growth.rate~TreatA)

summary(modelA)

# can we group RA and RB?

levels(TreatA)

TreatB<-TreatA

levels(TreatB)[3:4]<-'RA&RB'

levels(TreatB)

modelB<-lm(data.training$growth.rate~TreatB)

summary(modelB)

anova(modelA,modelB)

# model simplification justified.

# can we group Evolution and RA&RB?

TreatC<-TreatB

levels(TreatC)

levels(TreatC)[2:3]<-'E&RA&RB'

modelC<-lm(data.training$growth.rate~TreatC)

summary(modelC)

anova(modelB,modelC)

# model simplification justified.

# can we further group E&RA&RB and RC?

TreatD<-TreatC

levels(TreatD)

levels(TreatD)[2:3]<-'E&RA&RB&RC'

levels(TreatD)

modelD<-lm(data.training$growth.rate~TreatD)

summary(modelD)

anova(modelC,modelD)

# model C is the minimal adequate model.

tapply(data.training$growth.rate,TreatC,mean)

tapply(data.training$growth.rate,TreatC,sd)

# ANOVA analysis for infectivity data

tapply(data.training$infectivity.range,data.training$selection.treatment,mean)

tapply(data.training$infectivity.range,data.training$selection.treatment,sd)

Treat1<-as.factor(data.training$selection.treatment)

model1<-lm(data.training$infectivity.range~Treat1)

summary(model1)

# can we group RB and RC?

levels(Treat1)

Treat2<-Treat1

levels(Treat2)[4:5]<-'RB&RC'

levels(Treat2)

model2<-lm(data.training$infectivity.range~Treat2)

summary(model2)

anova(model1,model2)

# model simplification justified.

# can we group RA and RB&RC?

Treat3<-Treat2

levels(Treat3)

levels(Treat3)[3:4]<-'RA&RB&RC'

model3<-lm(data.training$infectivity.range~Treat3)

summary(model3)

anova(model2,model3)

# model simplification justified.

# can we further group Evolution and RA&RB&RC

Treat4<-Treat3

levels(Treat4)

levels(Treat4)[2:3]<-'E&RA&RB&RC'

levels(Treat4)

model4<-lm(data.training$infectivity.range~Treat4)

summary(model4)

anova(model3,model4)

# model simplification justified.

# model4 is the minimal adequate model.

tapply(data.training$infectivity.range,Treat4,mean)

tapply(data.training$infectivity.range,Treat4,sd)

# to detect outlier samples using PCA analysis

pca<-prcomp(x=data.frame(growth=data.training$growth.rate,infectivity=data.training$infectivity.range),scale=T,center=T,retx=T)

summary(pca)

biplot(pca)

pcs<-pca$x

apply(pcs,2,function(x) which(abs(x-median(x))>2*mad(x)))

apply(pcs,2,function(x) which(abs(x-median(x))>1.75*mad(x)))

data.training$phage.id[7]

data.training$phage.id[16]

data.training$phage.id[20]

data.training$phage.id[26]

# to detect outlier samples using linear regression

lm.1<-lm(infectivity.range~growth.rate,data=data.training)

summary(lm.1)

par(mfrow=c(2,2))

plot(lm.1)

data.training$phage.id[7]

data.training$phage.id[20]

data.training$phage.id[26]

lm.2<-lm(growth.rate~infectivity.range,data=data.training)

summary(lm.2)

par(mfrow=c(2,2))

plot(lm.2)

data.training$phage.id[2]

data.training$phage.id[16]

data.training$phage.id[20]

# multiple linear regression for how duration of control depends on growth and infectivity

model.duration<-lm(duration.control~growth.rate*infectivity.range,data=data.training)

library(car)

summary(model.duaration)

Anova(model.duration,type='2')

model.duration.2<-lm(duration.control~growth.rate+infectivity.range,data=data.training)

anova(model.duration,model.duration.2)

Anova(model.duration.2,type='2')

summary(model.duration.2)

Figure S1 Linear regression for the relationship between phage growth rate and infectivity range. Large-residual data points identified by model checking were annotated with their ID.


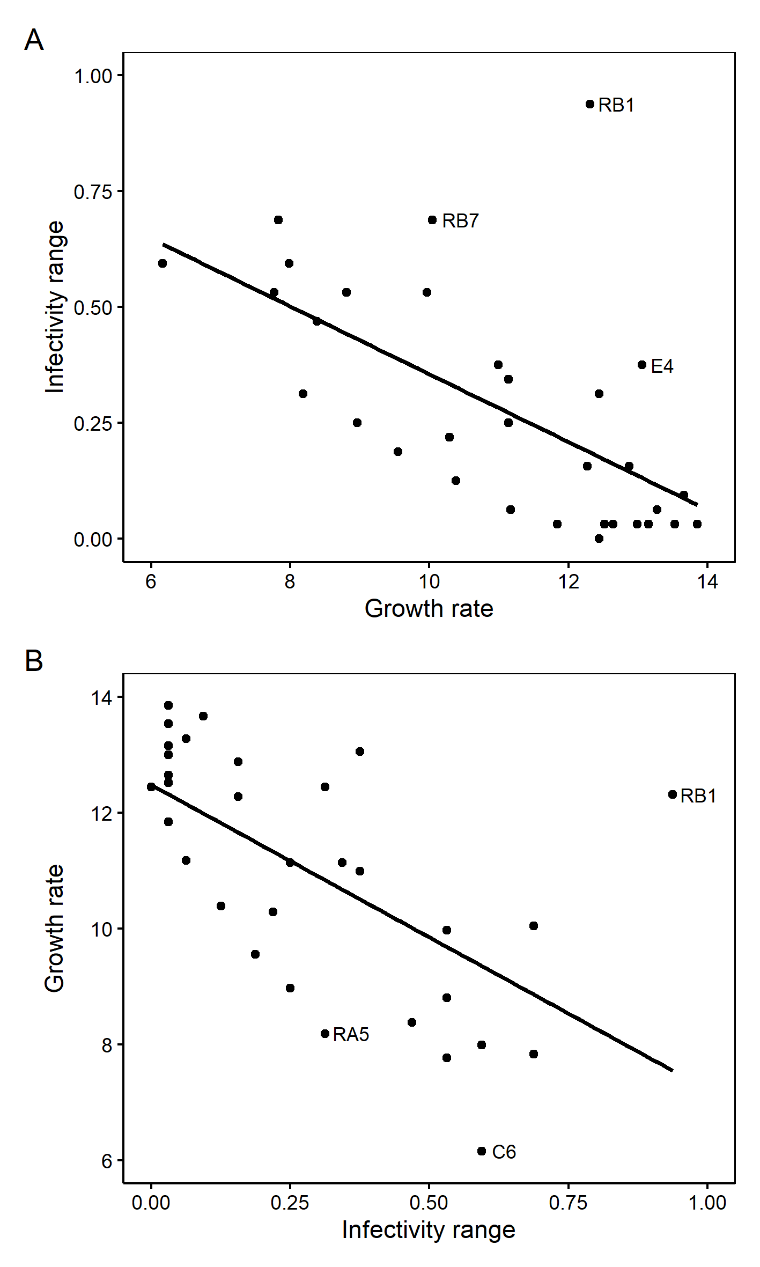


Table S1 Summary of growth rate and infectivity range data by selection treatment. ANOVA models for the differences among treatments were followed by stepwise model simplification, grouping treatment levels that did not differ significantly from each other. Treatment groups remaining in the minimal adequate models differed significantly from each other (*P* < 0.05).

| Growth rate | | | Infectivity range | | |
| --- | --- | --- | --- | --- | --- |
| Treatment | Sample size (n) | Mean ± SE | Treatment | Sample size (n) | Mean ± SE |
| Evolution | 8 | 12.0 ± 0.275 | Evolution | 8 | 0.180 ± 0.0581 |
| Coevolution | 3 | 7.25 ± 0.547 | Coevolution | 3 | 0.603 ± 0.0454 |
| Rotation A | 8 | 11.4 ± 0.811 | Rotation A | 8 | 0.210 ± 0.0772 |
| Rotation B | 8 | 11.8 ± 0.634 | Rotation B | 8 | 0.340 ± 0.119 |
| Rotation C | 5 | 9.81 ± 0.721 | Rotation C | 5 | 0.282 ± 0.0797 |
| *Groups of treatments in minimal adequate model of ANOVA* | | | | | |
| Evolution + Rotation A and B | 24 | 11.7 ± 0.343 | Evolution + Rotation A, B and C | 29 | 0.250 ± 0.0439 |
| Coevolution | 3 | 7.25 ± 0.547 | Coevolution | 3 | 0.603 ± 0.0454 |
| Rotation C | 5 | 9.81 ± 0.721 |  |  |  |

Table S2 Summary of genic-region mutations identified in the evolved phages. Numbers show each mutation’s total incidence in all phages and their incidences in phage groups categorized by growth, infectivity, or trade-off-overcoming, traits. Exact tests were used to analyze whether the occurrence of each mutation differed between phage groups (e.g., high- versus low-growth rate phages); and asterisks indicated where significant differences were observed (*, *P* < 0.05; **, *P* < 0.01; ***, *P* < 0.001).

| Gene | Gene function | Nucleotide site change | Amino acid change | Total hits in 32 phages | Hits in 16 high-growth phages | Hits in 16 broad-infectivity phages | Hits in 3 trade-off overcomers |
| --- | --- | --- | --- | --- | --- | --- | --- |
| *SBWP25_0002* | hypothetical protein | A1260C | No change, P | 1 | 1 | 1 | 0 |
|  |  | A1343G | E → G | 3 | 0 | 3 | 0 |
| *SBWP25_0003* | hypothetical protein | A1708G | No change, R | 1 | 1 | 1 | 1* |
| *SBWP25_0009* | hypothetical protein | 5919 1-bp deletion C | P → R (frameshifting) | 1 | 1 | 0 | 0 |
| *SBWP25_0014* | hypothetical protein | A9518G | No change, G | 1 | 0 | 1 | 0 |
| *SBWP25_0022* | hypothetical protein | 17862 9-bp deletion CGGCGGTTG | CCG deletion | 1 | 0 | 0 | 0 |
| *SBWP25_0024* | predicted phage RNA polymerase | C19391T | No change, C | 2 | 2 | 1 | 0 |
| *SBWP25_0027* | predicted phage virion structural protein | A22540G | Q → R | 2 | 2 | 2 | 1 |
|  |  | C22636T | T → I | 1 | 1 | 1 | 0 |
|  |  | 22695 3-bp insertion CGA | T insertion | 3 | 2 | 0 | 1 |
|  |  | 22695 3-bp insertion GGA | R insertion | 2 | 0 | 1 | 0 |
|  |  | 22698 3-bp insertion CGA | T insertion | 1 | 0 | 0 | 0 |
|  |  | 22699 6-bp insertion TAAGAA | NK insertion | 1 | 0 | 0 | 0 |
|  |  | 22699 3-bp insertion TAA | N insertion | 1 | 0 | 1 | 0 |
|  |  | 22701 3-bp insertion GGA | R insertion | 1 | 1 | 1 | 0 |
|  |  | 22702 6-bp insertion GAAGCA | KQ insertion | 2 | 0 | 2 | 0 |
|  |  | 22702 3-bp insertion GAA | K insertion | 2 | 1 | 1 | 0 |
|  |  | 22702 3-bp insertion GGA | E insertion | 1 | 0 | 1 | 0 |
|  |  | 22702 3-bp insertion GAA | K insertion | 1 | 1 | 0 | 0 |
|  |  | 22702 3-bp insertion GAT | I insertion | 1 | 0 | 1 | 0 |
|  |  | 22702 6-bp insertion GAAGAA | KK insertion | 1 | 0 | 1 | 0 |
|  |  | 22703 3-bp insertion AAA | K insertion | 1 | 1 | 0 | 0 |
| *SBWP25_0030* | predicted capsid protein | 25373 1-bp deletion A | M start-loss | 1 | 0 | 1 | 0 |
| *SBWP25_0032* | predicted phage tail tubular protein B | G27697A | A → T | 1 | 0 | 1 | 0 |
|  |  | T27764C | L → P | 1 | 0 | 1 | 0 |
|  |  | C27833T | A → V | 1 | 0 | 1 | 0 |
|  |  | G27835A | E → K | 1 | 0 | 1 | 0 |
|  |  | A27836C | E → A | 1 | 1 | 0 | 1* |
|  |  | A27836G | E → G | 2 | 2 | 1 | 0 |
|  |  | A27874C | K → Q | 1 | 0 | 0 | 0 |
|  |  | A28991G | K → R | 1 | 0 | 1 | 1* |
| *SBWP25_0036* | predicted phage tail fibre protein | A35898G | Q → R | 15 | 2*** | 11* | 2 |
|  |  | G36794A | A → T | 14 | 5 | 11** | 3* |
|  |  | C36861T | T → M | 2 | 1 | 0 | 0 |
|  |  | C36888A | T → K | 2 | 0 | 2 | 0 |
|  |  | A37265G | N → D | 1 | 1 | 0 | 0 |
|  |  | C37535A | S → Y | 4 | 0 | 2 | 0 |
|  |  | C37535T | S → F | 2 | 1 | 2 | 1 |
| *SBWP25_0043* | hypothetical phage repetitive protein | C42395T | H → Y | 1 | 0 | 1 | 0 |

**SI References**

Danovaro, R., Corinaldesi, C., Dell'Anno, A., Fuhrman, J. A., Middelburg, J. J., Noble, R. T., & Suttle, C. A. (2011). Marine viruses and global climate change. *FEMS Microbiology Reviews, 35*(6), 993-1034. doi:10.1111/j.1574-6976.2010.00258.x

Kassen, R. (2014). *Experimental evolution and the nature of biodiversity*. Greenwood Village, Colorado: Roberts and Company.

Lopez-Pascua, L., & Buckling, A. (2008). Increasing productivity accelerates host-parasite coevolution. *J. Evol. Biol., 21*(3), 853-860. doi:10.1111/j.1420-9101.2008.01501.x

Morgan, A. D., Gandon, S. G., & Buckling, A. (2005). The effect of migration on local adaptation in a coevolving host-parasite system. *Nature, 437*, 253-256.

Poullain, V., Gandon, S., Brockhurst, M. A., Buckling, A., & Hochberg, M. E. (2008). The evolution of specificity in evolving and coevolving antagonistic interactions between a bacteria and its phage. *Evolution, 62*(1), 1-11. doi:10.1111/j.1558-5646.2007.00260.x

Tuladhar, E., Bouwknegt, M., Zwietering, M. H., Koopmans, M., & Duizer, E. (2012). Thermal stability of structurally different viruses with proven or potential relevance to food safety. *Journal of Applied Microbiology, 112*(5), 1050-1057. doi:https://doi.org/10.1111/j.1365-2672.2012.05282.x

Ulmanen, I., Broni, B., & Krug, R. M. (1983). Influenza virus temperature-sensitive cap (m7GpppNm)-dependent endonuclease. *Journal of Virology, 45*(1), 27-35.

Zhang, Q.-G., & Buckling, A. (2011). Antagonistic coevolution limits population persistence of a virus in a thermally deteriorating environment. *Ecology Letters, 14*(3), 282-288.

Zhang, Q.-G., & Buckling, A. (2016). Resource-dependent antagonistic coevolution leads to a new paradox of enrichment. *Ecology, 97*(5), 1319-1328. doi:10.1890/15-1408.1
